# Primary Dentition Analysis: Exploring a Hidden Approach

**DOI:** 10.5005/jp-journals-10005-1323

**Published:** 2016-04-22

**Authors:** Sivakumar Nuvvula, Kalasandhya Vanjari, Rekhalakshmi Kamatham, Kumar Raja Gaddam

**Affiliations:** 1 Professor and Head, Department of Pedodontics and Preventive Dentistry Narayana Dental College, Nellore, Andhra Pradesh, India; 2 Postgraduate Student, Department of Pedodontics and Preventive Dentistry Narayana Dental College, Nellore, Andhra Pradesh, India; 3 Reader, Department of Pedodontics and Preventive Dentistry Narayana Dental College, Nellore, Andhra Pradesh, India; 4 Former Postgraduate Student, Department of Pedodontics and Preventive Dentistry Narayana Dental College, Nellore, Andhra Pradesh, India

**Keywords:** Analysis, BU approach, Primary dentition, Tanaka-Johnston.

## Abstract

**Background:** Accurate prediction of the mesiodistal widths (MDWs) of canines and premolars in children with primary dentition facilitates interception of malocclusion at an early age. Boston University (BU) approach is one, i.e., based on primary teeth for predicting canine and premolar dimensions.

**Aim:** To predict the canine and premolar dimensions, in the contemporary population, using BU approach and compare with the values obtained using Tanaka-Johnston (T/J) approach.

**Design:** Children in the age range of 7-11 years with presence of all permanent mandibular incisors and primary maxillary and mandibular canines and first molars were included in the study. Those with interproximal caries or restorations, abnormalities in shape or size and history of orthodontic treatment were excluded. Impressions of both arches were made using irreversible hydrocolloid and poured with dental stone. The MDWs of the required teeth were measured on the models using electronic digital vernier caliper from which widths of permanent canines and premolars were predicted using both T/J and BU approaches.

**Results:** Statistically significant (p = 0.00) positive correlation (r = 0.52-0.55) was observed between T/J and BU approaches. A statistically significant (p = 0.00) strong positive correlation (r = 0.72-0.77) was observed among girls, whereas boys showed a statistically nonsignificant weak positive correlation (r=0.17-0.42) based on gender.

**Conclusion:** Boston University approach can be further studied prospectively to make it possible as a prediction method of permanent tooth dimensions for children in primary dentition stage.

**How to cite this article:** Nuvvula S, Vanjari K, Kamatham R, Gaddam KR. Primary Dentition Analysis: Exploring a Hidden Approach. Int J Clin Pediatr Dent 2016;9(1):1-4.

## INTRODUCTION

The purpose of mixed dentition analysis (MDA) is to calculate the difference between the amount of the dental arch space available and that required for accommodating the permanent teeth for planning either preventive or interceptive approach. The available space in the arch can be equal to or greater/smaller than the unerupted teeth, which becomes fundamental in determining the treatment plan that might involve serial extractions, eruption guidance, space maintenance, space gain or simple monitoring of the occlusion.^1^

The methods employed for MDA can be grouped into three categories, i.e., the ones that use regression equations, those based on radiographs and a combination of both these approaches.^1-3^ Among the different MDA methods reported in the literature, those based on the regression equations are the most widely used, especially the Moyers’ probability tables and the Tanaka and Johnston (T/J) equations.^3^

The major drawback of these analyses is applicability only after the eruption of mandibular permanent incisors. Hence, Gianelly in his personal communication, proposed a prediction method, i.e., based on the mesiodistal widths (MDWs) of primary mandibular canines and first molars with an idea for early prediction of unerupted permanent mandibular teeth widths.^4^ This was presented in Boston University (BU), and hence the method was named as BU approach. Subsequently, two studies were carried out on this approach, one on Iowa population and the other on Iraqi population.^4,5^ Hence, the present study was conducted to test the validity of BU approach by comparing it with T/J approach in the contemporary population.

## METHODOLOGY

### Source of Data

Children in the age range of 7-11 years were selected from the schools of Nellore district (employing cluster sampling) after obtaining informed consent from the parents and the school authorities. The study was approved by the Institutional Ethical Committee and was ethically conducted in accordance with the Declaration of Helsinki.

### Method of Data Collection

Children with existence of primary maxillary and man-dibular canines and first molars and eruption of all permanent mandibular incisors were included. Those with interproximal caries or restorations, missing or supernumerary teeth, abnormalities in shape or size and history of orthodontic treatment were excluded.

Impressions of maxillary and mandibular arches were made using irreversible hydrocolloid (Tropicalgin, Chromatic alginate; Zhermack Spa, Italy), rinsed in running water, disinfected with 2% glutaraldehyde and poured with hard dental stone (Goldstone). The maximum MDWs of permanent mandibular incisors and primary maxillary and mandibular canines and first molars were measured using electronic digital vernier caliper (Aerospace 0-150 mm with a resolution of 0.01 mm), twice by two investigators (KV and RK). The MDWs of canines and premolars were predicted using both T/J and the considered BU approaches (described below) for all the children.

### Tanaka-Johnston Approach

- Mesiodistal widths of permanent maxillary canines and premolars = 11 + 0.5 (sum of MDWs of permanent mandibular incisors)
- Mesiodistal widths of permanent mandibular canines and premolars = 10.5 + 0.5 (sum of MDWs of permanent mandibular incisors).

### Boston University Approach

The original approach proposed by Gianelly is as follows: Mesiodistal widths of permanent mandibular canines and premolars = MDW of primary mandibular canine + 2 (MDW of primary mandibular first molar).

In the present study, we have extended the above formula by calculating the MDWs of both maxillary and mandibular primary canines and first molars for predicting the permanent tooth dimensions.

Thus, both MDW of primary mandibular canine + 2 (MDW of primary mandibular first molar) and MDW of primary maxillary canine + 2 (MDW of primary maxillary first molar) were calculated for both right and left sides and the obtained values were compared with those obtained through T/J approach.

### Statistical Analysis

Intra- and interrater reliability of both the investigators were obtained using Cohen’s kappa. The difference between the mean tooth dimensions and the predicted values obtained through all the above-mentioned methods in boys and girls was compared using unpaired t-test. The correlation between the methods was tested using Pearson correlation test with the level of significance set at 0.05. The mean values obtained through the BU and T/J approaches were compared using paired t-test for whole sample and also separately for boys and girls.

## RESULTS

Intrarater reliability values of first (KV) and second (RK) investigators were 0.96 and 0.94 respectively, whereas the interrater reliability was 0.91. The mean tooth dimensions of boys and girls are represented in Table 1, and the predicted values obtained through both the methods are presented in Table 2. When tooth dimensions of boys and girls were compared, the dimensions of all the teeth, except primary maxillary canines, differed statistically. The predicted values obtained through the considered methods also differed significantly between boys and girls. The correlations between the considered BU and T/J approaches are represented in Table 3 which depicts a statistically significant moderate positive correlation (r = 0.52-0.55). However, on segregating the data based on gender, a weak positive nonsignificant correlation was observed in boys (r = 0.17-0.42), whereas in girls there was a strong positive correlation that was statistically significant (r = 0.72-0.77). On comparing the difference between the mean values obtained through the BU and T/J approaches in boys and girls (Table 4), we observed a nonsignificant difference between BU approach (that employed mandibular primary teeth) and T/J for mandibular teeth in boys.

**Table 1:** **Table 1:** Mean values of individual tooth dimensions (tooth numbers in Federation Dentaire Internationale notation)

		*Tooth dimensions*					
		*Boys*		*Girls*			
*Tooth number*		*Mean± SD*		*Mean ± SD*		*p-value*	
54		7.35 ± 0.48		7.02 ± 0.47		0.02*	
53		6.83 ± 0.41		6.63 ± 0.45		0.11	
63		6.85 ± 0.42		6.62 ± 0.44		0.07	
64		7.33 ± 0.48		7.04 ± 0.45		0.04*	
74		8.10 ± 0.57		7.67 ± 0.45		0.01**	
73		6.00 ± 0.34		5.78 ± 0.36		0.03*	
32		6.27 ± 0.31		5.97 ± 0.37		0.003**	
31		5.65 ± 0.34		5.38 ± 0.34		0.01**	
41		5.65 ± 0.33		5.96 ± 0.38		0.01**	
42		6.26 ± 0.33		5.96 ± 0.38		0.01**	
83		5.99 ± 0.35		5.78 ± 0.33		0.04*	
84		8.12 ± 0.55		7.66 ± 0.45		0.003**	

*Significant at 0.05 level; **Significant at 0.01 level; SD: Standard deviation

**Table 2:** **Table 2:** Predicted permanent canine and premolar dimensions using various approaches

*Method employed*		*Estimated permanent canine and premolars dimension*				*p-value*	
		*Mean± SD*					
		*Boys (n = 26)*		*Girls (n = 23)*		*(Boys vs girls)*	
T/J (Lower)		22.41 ± 0.62		21.85 ± 0.67		0.004**	
T/J (Upper)		22.91 ± 0.62		22.35 ± 0.67		0.004**	
BU (Right upper)		21.53 ± 1.17		20.68 ± 1.30		0.02*	
BU (Left upper)		21.51 ± 1.16		20.71 ± 1.25		0.03*	
BU (Left lower)		22.20 ± 1.41		21.11 ± 1.15		0.005**	
BU (Right lower)		22.22 ± 1.39		21.11 ± 1.10		0.003**	

*Significant at 0.05 level; **Significant at 0.01 level; T/J: Tanaka-Johnston; BU: Boston University; N: Sample size; SD: Standard deviation

**Table 3:** **Table 3:** Correlations between T/J and BU approaches

				*Total sample (n = 49)*				*Boys (n= 26)*				*Girls (n = 23)*			
*Method employed*				*T/J (Lower)*		*T/J (Upper)*		*T/J (Lower)*		*T/J (Upper)*		*T/J (Lower)*		*T/J (Upper)*	
BU (RU)		r		0.55		0.55		0.23		0.23		0.72		0.72	
		P		0.00**		0.00**		0.26		0.26		0.00**		0.00**	
BU (LU)		r		0.53		0.53		0.2		0.2		0.72		0.72	
		P		0.00**		0.00**		0.33		0.33		0.00**		0.00**	
BU (LL)		r		0.52		0.52		0.2		0.2		0.73		0.73	
		P		0.00**		0.00**		0.32		0.32		0.00**		0.00**	
BU (RL)		r		0.52		0.52		0.17		0.17		0.77		0.77	
		P		0.00**		0.00**		0.42		0.42		0.00**		0.00**	

*Significant at 0.05 level; **Significant at 0.01 level; RU: Right upper; LU: Left upper; LL: Left lower; RL: Right lower; N: Sample size; r: Correlation coefficient; T/J: Tanaka-Johnston; BU: Boston University

**Table 4:** **Table 4:** Difference between T/J *vs* BU approaches

		*p-value*					
		*Total sample*		*Boys*		*Girls*	
*Method employed*		*(n = 49)*		*(n = 26)*		*(n = 23)*	
T/J (Lower) *vs* BU (RU)		0.00**		0.001**		0.00**	
T/J (Lower) *vs* BU (LU)		0.00**		0.001**		0.00**	
T/J (Lower) *vs* BU (LL)		0.01**		0.46		0.00**	
T/J (Lower) *vs* BU (RL)		0.01**		0.5		0.00**	
T/J (Upper) *vs* BU (RU)		0.00**		0.00**		0.00**	
T/J (Upper) *vs* BU (LU)		0.00**		0.00**		0.00**	
T/J (Upper) *vs* BU (LL)		0.00**		0.02*		0.00**	
T/J (Upper) *vs* BU (RL)		0.00**		0.02*		0.00**	

*Significant at 0.05 level; **Significant at 0.01 level; RU: Right upper; LU: Left upper; LL: Left lower; RL: Right lower; N: Sample size; T/J: Tanaka-Johnston; BU: Boston University

## DISCUSSION

In the present study, BU approach was revisited, as the prediction of MDWs of permanent canines and premolars can be accomplished even when the child is in the primary dentition stage. For comparison, T/J approach was considered as it is an acceptable method universally and does not require any specialized armamentarium, such as radiographs or prediction tables. Moreover, T/J approach was the one that was used for comparison in the previous studies on BU method.^4,5^

In the Iowa study, the correlation of actual tooth dimensions with those predicted using T/J was found to be 0.59, whereas that with BU method was 0.39.^4^ In another study on Iraqi population, there was 0.17 correlation of T/J and 0.22 correlation of BU approach with the original teeth dimensions.^5^ In the present study, the children studied were still in mixed dentition and the study design was cross-sectional; hence, we could not compare the predicted values with the original ones. This is the major limitation of the present study, though we are planning to prospectively follow the children to get the original dimensions as a further study. On comparing the predicted values with the considered BU and T/J approaches, we observed correlation coefficients in the range of 0.52-0.55. However, on segregating the data into boys and girls, the correlation was strong among girls (r = 0.72-0.77) when compared with boys (r = 0.17-0.42), which is in agreement with a previous study stating a better correlation of BU approach for girls.^5^

Gender differences in the prediction values are well-reported, because of the differences in the individual tooth dimensions; boys reported to have larger mesio-distal diameters when compared with girls.^1,4-6^ In the present study too, boys had larger dimensions for all the considered teeth, with statistical nonsignificance noted only for the maxillary primary canines. Concor-dantly, the predicted values obtained through all the considered approaches were larger in boys when compared to girls.

The mean predicted values obtained through T/J approach were larger when compared to all the considered BU approaches, which is in harmony with other studies.^4,5^ As we could not compare the predicted values with the original dimensions, we cannot comment on the overestimation or underestimation of the approaches employed. However, studies that tested the applicability of T/J approach in Indian population reported an overestimation of the unerupted teeth size.^7,8^ Also previous studies have reported that BU approach underestimated, whereas T/J approach overestimated the unerupted teeth dimensions in their respective population.^4,5^

On observing the correlations in the present study, it seems as if the BU approach cannot be suggested in boys. However, the comparison of the mean values obtained through the BU and T/J approaches showed an encouraging point. We found a nonsignificant difference between T/J values for mandibular teeth and predicted values obtained through BU using primary mandibular teeth in boys, which is actually the original BU approach proposed (Table 4). Though there was no linear correlation between BU and T/J approaches among boys, not much difference was observed in the mean predicted values for the mandibular teeth with both the approaches.

The limitation for using primary dentition analysis is that the changes in arch dimensions as well as tooth position and inclination that maintain the balance among the various functional and structural demands placed on the face and dentition are difficult to predict in an early age. Due to this reason, some hesitate to recommend BU approach, but not that it can/cannot predict the tooth size.^9^

## CONCLUSION

In spite of the limitations, we recommend the use of BU approach to predict arch length tooth material discrepancy at an early age, to get at least an approximate estimation of the required space. We also advocate the necessity of further research on this approach pro-spectively.
